# Micro/nanoplastics induce thyroid follicular cell pyroptosis to trigger thyrotoxicity by activating NF-κB signaling

**DOI:** 10.1080/07853890.2026.2624175

**Published:** 2026-02-17

**Authors:** Fangda Fu, Yuying Chen, Huan Luo, Hongfeng Ruan

**Affiliations:** ^a^ Institute of Orthopaedics and Traumatology, The First Affiliated Hospital of Zhejiang Chinese Medical University (Zhejiang Provincial Hospital of Traditional Chinese Medicine), Hangzhou, PR China; ^b^ The Fourth Clinical Medical College of Zhejiang Chinese Medical University, Hangzhou, PR China; ^c^ Department of Pharmacy, The Second Affiliated Hospital, School of Medicine, Zhejiang University, Hangzhou, PR China

**Keywords:** Micro/nanoplastics, NF-κB signaling pathway, NLRP3 inflammasome, pyroptosis, thyroid toxicity

## Abstract

**Background:**

Micro/nanoplastics (MNP) have emerged as ubiquitous environmental contaminants with demonstrated bioaccumulation potential in organisms through multiple exposure pathways, posing substantial health risks globally. While mounting evidence indicates that MNP exposure adversely affects various organ systems including the nervous, reproductive, and digestive systems, the specific mechanisms underlying MNP-induced thyrotoxicity remain enigmatic.

**Methods:**

4-week-old male C57BL/6 mice were administered microplastics (MP, 5 μm) or nanoplastics (NP, 50 nm) *via* intragastric gavage at 30 mg/kg for 4 and 8 weeks. The thyroid architecture and endocrine function were evaluated by histological staining and thyroid hormones ELISA kit. The expression of apoptosis indicators (BCL2, BAX, CASPASE3), inflammatory factors (IL-1β, IL-18, TNF-α) and pyroptosis related-proteins (NLRP3, CASPASE1 and GSDMD), as well as the activity of NF-κB signaling were determined by immunofluorescence.

**Results:**

We found that MNP exposure induces significant thyrotoxicity characterized by disrupted thyroid follicular architecture, comprised endocrine function, heightened apoptosis, and excessive inflammatory cytokines production, with NP exhibiting a more pronounced effect than MP. Mechanistically, MNP exposure stimulated thyroid follicular cell pyroptosis by upregulation of key pyroptotic mediators including NLRP3, CASPASE1, and GSDMD, driven by NF-κB signaling pathway activation.

**Conclusion:**

Collectively, these findings provide novel mechanistic insights into MNP-induced thyroid toxicity and highlight the critical role of follicular cell pyroptosis, contributing to our understanding of the adverse health consequences associated with environmental plastic pollution.

## Introduction

Global plastic production has experienced unprecedented exponential growth, escalating to over 8,300 megatons in recent decades [1]. Conventional plastics fragment is degraded into microplastics (MP; 1 µm-5 mm diameter) or nanoplastics (NP; <1 µm diameter) through different environmental degradation processes, which can bioaccumulate through multiple exposure routes, especially dietary ingestion, posing significant environmental and health concerns due to their persistence and limited biodegradability [2,3]. Accumulating toxicological evidence demonstrates that MNP can traverse critical biological barriers, especially traverse the gastrointestinal barrier into circulatory system, leading to systemic distribution and organ-specific accumulation [4–10], underscoring the potential for widespread organ system dysfunction.

The thyroid gland, as the largest endocrine organ, plays a fundamental role in physiological homeostasis through the secretion of thyroid hormones, primarily thyroxine (T_4_) and triiodothyronine (T_3_), which regulate cellular metabolism, growth, and developmental processes by their unbound forms, free T_3_ (fT_3_) and free T_4_ (fT_4_) [11,12]. Thyroid hormone synthesis occurs within follicular epithelial cells and is stored in colloid-filled follicles, therefore, the morphology especially follicle diameter and structural integrity, directly correlates with thyroid functional status [13]. This relationship is exemplified in autoimmune thyroid disorders such as Hashimoto thyroiditis, where characteristic alterations in follicular architecture accompany endocrine dysfunction [14]. Besides, thyroid function is precisely regulated through the hypothalamic-pituitary-thyroid axis *via* thyrotropin (TSH) feedback mechanisms [15].

The thyroid’s extensive vascularization renders it particularly susceptible to blood-borne environmental contaminants and toxicants, including MNP. Supporting this susceptibility, previous studies demonstrate significant MNP tissue accumulation following systemic exposure, with detectable concentrations across multiple organs including blood (135.86 ± 7.2 μg/mL) and various tissues, confirming the potential for thyroid bioaccumulation through circulatory distribution [16]. These contaminants can disrupt thyroid cellular integrity and compromise glandular function, potentially leading to endocrine dysfunction and thyroid pathology [12,17]. Previous studies have documented thyroid-disrupting effects of plastic particle exposure: 100 μg/L NP (70 nm) induced thyroid dysfunction in zebrafish, characterized by reduced T_3_ and T_4_ levels [18]. Similarly, 5-week exposure to NP (25 and 50 nm) significantly decreased fT_3_ levels and elevated TSH concentrations in adult male Wistar rats, confirming MNP-induced thyroid impairment [19]. Nevertheless, the precise molecular mechanisms underlying MNP-induced thyrotoxicity remain completely clarified.

Pyroptosis represents a distinct form of inflammatory programmed cell death characterized by the activation of the NOD-like receptor protein 3 (NLRP3) inflammasome complex [20]. Upon activation, this multiprotein complex facilitates the cleavage of pro-CASPASE1 to active form, which subsequently processes gasdermin-D (GSDMD) to form plasma membrane pores and promotes pro-inflammatory cytokines interleukin 1β (IL-1β) and interleukin 18 (IL-18) that enable pyroptotic cell death [21]. Emerging evidence implicates MNP-induced pyroptosis in multi-organ toxicity: 5 µm MP exposure activated pyroptosis in liver, heart, lung, and colon in both murine and avian models, leading to extensive inflammatory infiltration and structural-functional impairment [22–25]. Moreover, 60-day exposure to 0.5 ppm NP (500 nm) worsened hepatic damage in male Kunming mice through NLRP3/CASPASE1-mediated pyroptosis activation [26]. Our recent findings demonstrated that cadmium exposure compromised thyroid follicular structure and endocrine function, accompanied by significant upregulation of key pyroptosis mediators including NLRP3, Apoptosis-associated speck-like protein, GSDMD, and CASPASE1 [12]. These observations suggest that pyroptosis may serve as a critical mechanism mediating environmental pollutant-induced thyroid dysfunction, leading to the hypothesis that MNP exposure may similarly trigger thyroid follicular cell pyroptosis.

The nuclear factor-kappa B (NF-κB) signaling pathway has been identified as a key regulator of thyroid cell pyroptosis induced by environmental stressors, orchestrating inflammatory cytokine production, apoptosis, thyroid structural damage, and endocrine dysfunction [12,27–29]. Accumulating evidence suggests that MNP treatment significantly activates NF-κB signaling pathway across multiple biological systems [24,25,30]. For instance, 35-day MP exposure in ICR male mice resulted in marked NF-κB upregulation accompanied by elevated levels of inflammatory factors, (IL-1β and IL-6) in sperm, leading to increased sperm abnormalities and reproductive toxicity [31]. Similarly, 28-day exposure to 5 μm MP in C57BL/6 J mice induced oxidative stress, colonic inflammatory infiltration, and intestinal barrier dysfunction through NF-κB/NLRP3/IL-1β axis activation [24]. However, whether MNP exposure modulates thyroid follicular cell pyroptosis to exacerbate thyroid damage through NF-κB signaling remains unexplored.

Given the widespread environmental distribution of MNP and the mounting evidence of their toxicological potential, this study aimed to investigate whether exposure to environmentally relevant microplastics induces thyroid follicular cell pyroptosis *via* the NF-κB signaling pathway. To accomplish this goal, 36 healthy male C57BL/6 mice (4 weeks old) were randomly allocated into 3 experimental groups and intragastrically administered with normal saline (control), MP (5 μm in diameter), and NP (50 nm in diameter) at 30 mg/kg body weight (five times weekly for 4 and 8 weeks). Comprehensive assessment included serum thyroid hormone levels, histopathological evaluation of thyroid architecture, and potential molecular alterations through ELISA, immunofluorescence (IF) analysis, etc. Our findings provide novel mechanistic insights into MNP-induced thyrotoxicity and contribute to the understanding of plastic particle-mediated endocrine disruption.

## Material and methods

### Chemicals and reagents

MNP, comprising 5 μm MP (polydispersity index:0.326; Zeta potential: −19.78 mV) and 50 nm NP (polydispersity index:0.281; Zeta potential: −24.49 mV), were purchased from Rigor Science Corp. (Wuxi, Jiangsu, China) (Figure S1). ELISA kits for free Triiodothyronine assay kit (fT_3_, H222), Thyroxine (fT_4_, H223), Thyrotropin (TSH, H087-1-2), and Interleukin-1β (IL-1β, H002) were sourced from Nanjing Jiancheng Bioengineering Institute (Nanjing, China). Hematoxylin–Eosin (HE) staining kit and Periodic Acid-Schiff (PAS, GLYCOPRO-1KT) staining kit were from Solarbio (Beijing, China). Primary antibodies used in this study are listed in Table 1. Fluorescent secondary antibody was provided by Sungene Biotech Co. (Tianjin, China). Vector^®^ TrueVIEW^®^ Autofluorescence Quenching Kit (SP-8500-15) with DAPI was purchased from Vector Laboratories Inc (Newark, USA). TUNEL Bright Green Apoptosis Detection Kit (A112-01) was acquired from Vazyme Biotech (Nanjing, China). Unless specified, all chemicals were supplied by Sigma-Aldrich (St. Louis, MO, USA).

**Table 1.** Detailed information of antibodies used in this study.

Antibody	Host species	Dilution	Catalog	Company
BAX	Rabbit	1:300	RLM3619	Ruiying biological
BCL2	Rabbit	1:300	RLM3401	Ruiying biological
CASPASE3	Rabbit	1:500	RLM3431	Ruiying biological
IL-1β	Rabbit	1:400	RLT4001	Ruiying biological
IL-18	Rabbit	1:400	RLN1926	Ruiying biological
TNF-α	Rabbit	1:400	RLT4689	Ruiying biological
NLRP3	Rabbit	1:400	19771-1-AP	Proteintech
GSDMD	Rabbit	1:400	ab219800	Abcam
CASPASE1	Rabbit	1:400	22915-1-AP	Proteintech
NF-Κb	Rabbit	1:500	8242 S	Cell Signaling Technology
p-NF-κB	Rabbit	1:500	RLP0191	Ruiying biological

### Animals and treatment

Male C57BL/6 mice (*n* = 36, 4 weeks old, 14–16 g) were obtained from the animal experiments center of Zhejiang Chinese Medical University (Grade SPF, SCXK (Shanghai)) and housed in a specific pathogen-free barrier system maintained at 23 ± 2 °C with 50 ± 5% relative humidity under a 12-h light/dark cycle. All animals had unrestricted access to food (Shenyang Maohua Biotechnology Co., Ltd., Shenyang Province, China) and water. All experimental protocols for mice were approved by the Committee on the Ethics of Animal Experiments of Zhejiang Chinese Medical University (NO.20220103-02) and conducted in compliance with the Guide for the Care and Use of Laboratory Animals (NIH Publication, 8th Edition, 2011).

Following a 1-week acclimatization period, all mice were randomly divided into 3 groups and assigned to random cages (*n* = 12 per group): Control group (normal saline), MP group (30 mg/kg MP), and NP group (30 mg/kg NP). MNP suspensions were freshly prepared in normal saline and administered *via* intragastric gavage 5 times per week for 4 or 8 weeks. The sample size and the dosage of MNP were chosen based on previous studies [12,16,32,33]. At the designated time points (4 and 8 weeks), mice were anesthetized with an overdose of 2% sodium pentobarbital (40 mg/kg, i.p.) with no animals regained consciousness following AVMA guidelines. Blood samples were collected *via* abdominal aortic puncture. Serum was separated by centrifugation at 1000*g* for 10 min at room temperature and stored at −80 °C until analysis. Thyroid samples were carefully dissected and processed for further analysis, as described previously [12,28]. All experimental animals completed treatment and subsequent sample collection, with no deaths, health deterioration, or data exclusion from the analysis during the study. The order of measurements was randomized across groups to prevent systematic bias. The investigators performing the outcome assessment and data analysis were blinded to the group allocation.

### Serum biochemical analysis

Serum concentrations of thyroid hormones (fT_3_, fT_4_, and TSH) and the inflammatory cytokine IL-1β were determined using commercially available ELISA kits, following the manufacturer’s instructions.

### Histological analysis

Thyroid tissue was fixed in 4% paraformaldehyde for 24 h at 4 °C, dehydrated in 30% sucrose solution overnight, embedded in OCT compound (Sakura Finetek, Japan), and sectioned at 8 µm thickness. Histological examination was performed using H&E and PAS staining according to standard protocols. Digital images were captured using light microscopy (Carl Zeiss, Gottingen, Germany). Morphometric analysis was conducted using Image-Pro Plus Software 6.0 (Media Cybernetics Inc., Rockville, MD, USA). The diameter of the follicle (DF) was calculated by averaging measurements along four directional axes through the follicle center (0°, 45°, 90°, 135°): DF = (d1 + d2 + d3 + d4)/4, as previously described [12,28]. Follicular epithelial cell thickness and follicle number per 0.2 mm^2^ were quantified from at least six randomly selected fields per section.

Histopathological scoring was performed by a blinded observer using established criteria [34], evaluating: (1) loss of zonal variation, (2) thyrocytes cytoplasmic vacuolization, (3) scalloping and random colloid vacuolization, (4) papillary budging, (5) cystic degeneration, and (6) chronic thyroiditis.

### IF analysis

Sections were permeabilized, blocked with 5% normal goat serum, and incubated overnight at 4 °C with primary antibodies against apoptosis markers (BAX, BCL2, CASPASE3), inflammatory cytokines (IL-1β, IL-18, TNF-α), pyroptosis markers (NLRP3, GSDMD, CASPASE1), and NF-κB signaling components (NF-κB, and p-NF-κB). Following primary antibody incubation, sections were incubated with fluorescent-conjugated secondary antibody for 1 h at 37 °C in the dark. Nonspecific fluorescence was quenched using Vector^®^ TrueVIEW^®^ Autofluorescence Quenching Kit. Fluorescent images were acquired using a fluorescence microscope (Carl Zeiss, Gottingen, Germany) with consistent exposure settings across all samples. IF intensity was quantified using Image-Pro Plus Software, with integrated optical density values calculated from regions of interest by a blinded observer. At least six randomly selected fields per section were analyzed, and the mean fluorescence intensity was normalized to the control group.

### TUNEL assay

DNA fragmentation was detected using the TUNEL Bright Green Apoptosis Detection Kit following the manufacturer’s instructions. Briefly, sections were permeabilized and incubated with terminal deoxynucleotidyl transferase (TdT) enzyme solution for 1 h at 37 °C. Negative controls were by omitting the TdT enzyme and substituting with PBS. Cell nuclei were counterstained with DAPI, and TUNEL-positive cells were identified by green fluorescence. The percentage of apoptotic cells was calculated by counting TUNEL-positive cells relative to the total number of DAPI-stained nuclei in at least six randomly selected fields per section by a blinded observer.

### Statistics analysis

All data are presented as mean ± standard deviation (SD). Statistical analyses were performed using GraphPad Prism 8 statistics software (GraphPad by Dotmatics, California, USA). Comparisons between two groups were assessed using a two-tailed, unpaired Student’ *t* test, while multiple group comparisons were analyzed using one-way or two-way ANOVA followed by Tukey’s post hoc test for multiple comparisons. Statistical significance was considered for *p* value <0.05.

## Results

### MNP exposure induces systemic toxicity in mice

**Figure 1.**
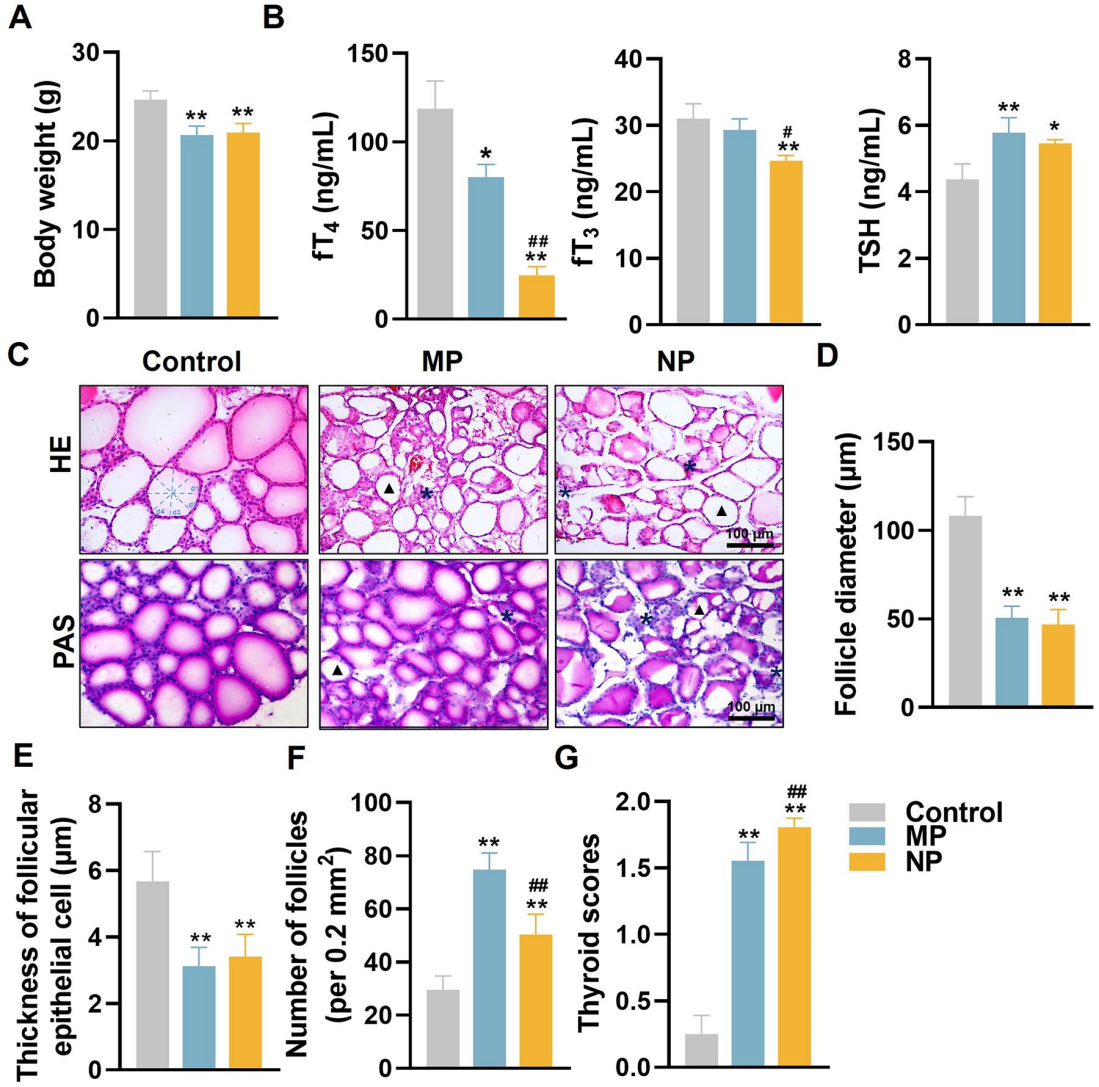
MNP exposure disrupts thyroid structure and function in mice. (A) Body weight measurements in control and MNP exposure mice after 8 weeks of treatment. (B) Serum concentrations of fT_4_, fT_3_, and TSH were determined by ELISA following 8-week MNP exposure. (C) Representative histological images of thyroid sections with HE and PAS staining showing thyroid morphological changes in control and MNP-exposed mice at 8 weeks. Black triangle indicates the cytoplasmic vacuolization in thyroid follicles; black (*) mark inflammatory cell infiltration in thyroid tissue. (D) Quantification analysis of thyroid follicle diameter (DF), calculated by averaging measurements along four directional axes through the follicle center (0°, 45°, 90°, 135°). DF = (d1 + d2 + d3 + d4)/4. (E) Quantification of follicular epithelial cell thickness. (F) Quantification of number of follicles per 0.2 mm^2^ area. (G) Histopathological scoring of thyroids follicular architecture. Data are presented as mean ± SD (*n* = 6 per group). *p < 0.05, **p < 0.01 (compared with control group), ^#^*p* < 0.05, ^##^*p* < 0.01 (compared with MP group).

To comprehensively evaluate the subchronic toxicity of MNP exposure on the entire organism, we initially monitored body weight changes in mice following 8 weeks of MNP exposure. Both MP and NP treatments resulted in significant reductions in body weight compared to the control mice (Figure 1A). However, no significant difference was observed between NP and MP groups, suggesting that the systemic toxic effects were independent of particle size under the tested conditions.

### MNP exposure disrupts thyroid architecture and endocrine function

To assess the impact of MNP exposure on thyroid function, we analyzed serum levels of thyroid hormones (fT_3_, fT_4_, and TSH). As shown in Figure 1B, MNP exposure significantly disrupted thyroid hormone homeostasis. The most pronounced effect was observed in fT_4_ levels, where MP treatment reduced serum concentrations to 80.08 ± 7.18 ng/mL, compared to 118.70 ± 15.60 ng/mL in controls (32.5% reduction), while NP exposure caused an even more dramatic decrease to 24.72 ± 4.88 ng/mL, representing a 79.2% reduction from control levels and significantly lower than the MP group. While MP exposure did not significantly alter fT_3_ levels (29.31 ± 1.67 ng/mL *vs.* 31.03 ± 2.23 ng/mL in controls), NP treatment resulted in a significant reduction in serum fT_3_ (24.66 ± 0.82 ng/mL), which was significantly lower than both control and MP-treated groups. Consequently, both treatments triggered compensatory elevation of TSH levels through negative feedback mechanisms, with MP and NP groups showing 32.0% and 24.7% increases, respectively (5.78 ± 0.45 ng/mL and 5.46 ± 0.10 ng/mL, *vs.* 4.38 ± 0.46 ng/mL in controls). These findings demonstrate particle size-dependent thyroid disruption, with NP exhibiting substantially more potent thyroid-suppressive effects than MP.

Histological assessment using H&E and PAS staining revealed progressive morphological deterioration in thyroid architecture following MNP exposure (Figure 1C). Control mice displayed well-organized thyroid follicles with characteristic round-to-oval morphology, intact follicular epithelium, and homogeneous colloid distribution (follicle diameter: 108.2 ± 10.8 μm; follicular epithelial cell thickness: 5.7 ± 0.9 μm; follicle density: 29.5 ± 5.2 per 0.2mm^2^) (Figure 1D–F). Both MP and NP treatments induced significant alterations, including marked reductions in follicle diameter (50.7 ± 6.5 μm and 46.8 ± 8.4 μm, respectively) and epithelial cell thickness (3.1 ± 0.6 μm and 3.4 ± 0.7 μm), representing approximately a 53–57% and 46–40% decrease compared to controls (Figure 1D–E); Concomitantly, follicle density increased substantially in both treatment groups (74.8 ± 6.2 per 0.2 mm^2^ and 50.3 ± 7.7 per 0.2 mm^2^, respectively) (Figure 1F), indicative of follicular fragmentation and compensatory hyperplasia. Qualitative assessment revealed distinct pathological patterns: MP-treated thyroid tissue exhibited irregular follicular configurations, cytoplasmic vacuolization (marked by black triangles), moderately increased interfollicular spacing, and focal inflammatory infiltrates (marked by black *) (Figure 1C). NP exposure resulted in more severe architectural disruption, characterized by extensive follicular deformation, pronounced colloid vacuolization, tissue collapse with structural tears, and widespread inflammatory infiltration (Figure 1C). Histopathological scoring confirmed significantly greater tissue damage in both treatment groups, with NP showing more severe overall pathology than MP (Figure 1G).

### MNP exposure induces apoptosis in thyroid follicular cells

**Figure 2.**
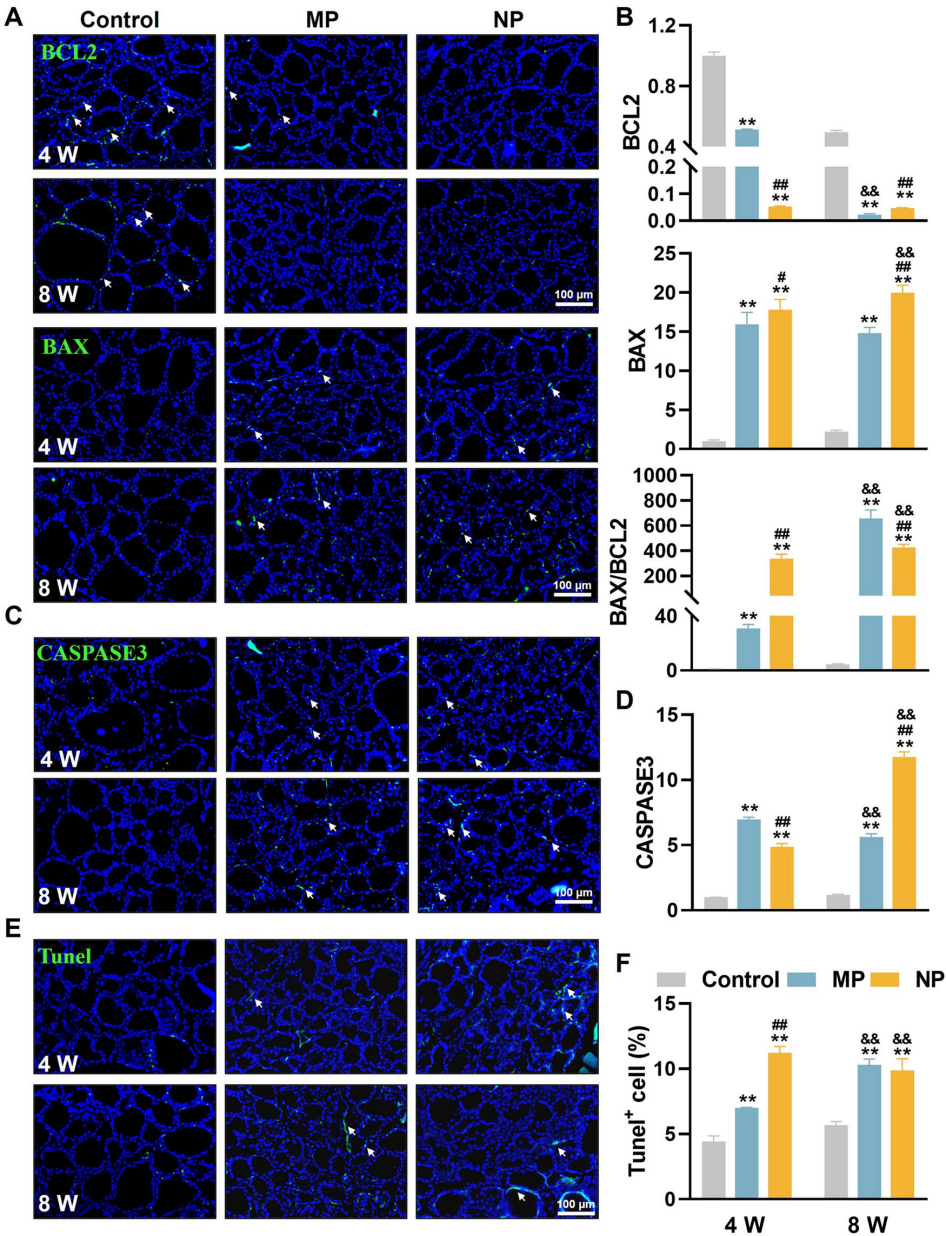
MNP exposure induces apoptosis in thyroid tissue of mice. (A) Representative IF images showing BCL2 and BAX expression (green) in thyroid at 4- and 8 weeks of MNP exposure. (B) Quantification analysis of BCL2 and BAX positive cells and the corresponding BAX/BCL2 ratio. (C) IF staining demonstrating CASPASE3 expression (green) in thyroid tissue following 4 and 8 weeks of MNP exposure. (D) Quantification of CASPASE3 positive cells. (E) Tunel staining to detect apoptosis cells in thyroid sections after 4 and 8 weeks of MNP exposure. (F) Quantitative analysis of Tunel-positive cell percentage. All sections were counterstained with DAPI (blue) to visualize cell nuclei. White arrows indicate positive expression in thyroid tissue. Data are presented as mean ± SD (*n* = 6 per group). **p < 0.01 (versus control group at the same exposure time point); ^#^*p* < 0.05, ^##^*p* < 0.01 (versus MP group at the same exposure time point); ^&&^*p* < 0.01 (versus 4-week exposure group within the same treatment group).

Given the observed structural damage and inflammatory responses in thyroid tissue, we next examined whether MNP exposure triggers apoptotic cell death in thyroid follicular cells. IF analysis was performed to assess the expression of key apoptotic regulators, including the anti-apoptotic protein BCL-2, pro-apoptotic protein BAX, and the apoptotic executioner cleaved CASPASE3, at both 4 and 8 weeks post-exposure. We found that MNP exposure significantly disrupted the balance between pro- and anti-apoptotic proteins in thyroid follicular cells. BCL-2 expression was progressively downregulated in both MP and NP treatment groups across both time points, indicating compromised cellular survival signaling. Conversely, BAX expression showed corresponding upregulation, with the exception of a modest decrease in the MP group at 8 weeks compared to 4 weeks (Figure 2A,B). This reciprocal regulation resulted in a significant time-dependent increase in the BAX/BCL-2 ratio for both treatments, reflecting enhanced apoptotic susceptibility of thyroid follicular cells following MNP exposure. Consistently, CASPASE3 demonstrated marked elevation in both treatment groups (Figure 2C). At the 4-week time point, MP and NP treatments induced 7.0-fold and 4.9-fold increases in CASPASE3, respectively. Notably, differential temporal patterns emerged by 8 weeks: while MP-induced CASPASE3 levels showed a slight decrease to 5.6-fold, NP treatment resulted in a dramatic escalation to 11.8-fold above control levels. This divergent pattern suggests that NP exposure induces more sustained and progressive apoptotic activation compared to MP treatment (Figure 2D).

To validate the biochemical evidence of apoptosis, TUNEL staining was employed to detect DNA fragmentation, a hallmark of apoptotic cell death, in thyroid follicular cells (Figure 2E,F). Quantitative analysis revealed a clear progression of apoptotic cell death following MNP exposure. At 4 weeks, TUNEL-positive cells increased from 4.4% in control thyroid tissue to 7.0% in the MP group and 11.2% in the NP group, demonstrating both treatments’ capacity to induce apoptosis, with NP showing superior potency. By 8 weeks, an interesting convergence pattern emerged, with both MP and NP treatment groups showing approximately 10% TUNEL-positive cells, suggesting that prolonged exposure leads to similar levels of apoptotic cell death regardless of particle size. This temporal convergence, combined with the sustained elevation of apoptotic markers, indicates that MNP exposure creates a chronic apoptotic burden in thyroid follicular cells. Collectively, these findings establish that both MP and NP induce significant apoptotic cell death in thyroid follicular cells. NP demonstrating enhanced apoptotic responses in the early stage while MP took longer to accumulate to a similar level of apoptotic response. This evidence may contribute to the more severe thyroid dysfunction observed with NP exposure.

### Exposure to MNP stimulates inflammatory infiltration in thyroid tissues

**Figure 3.**
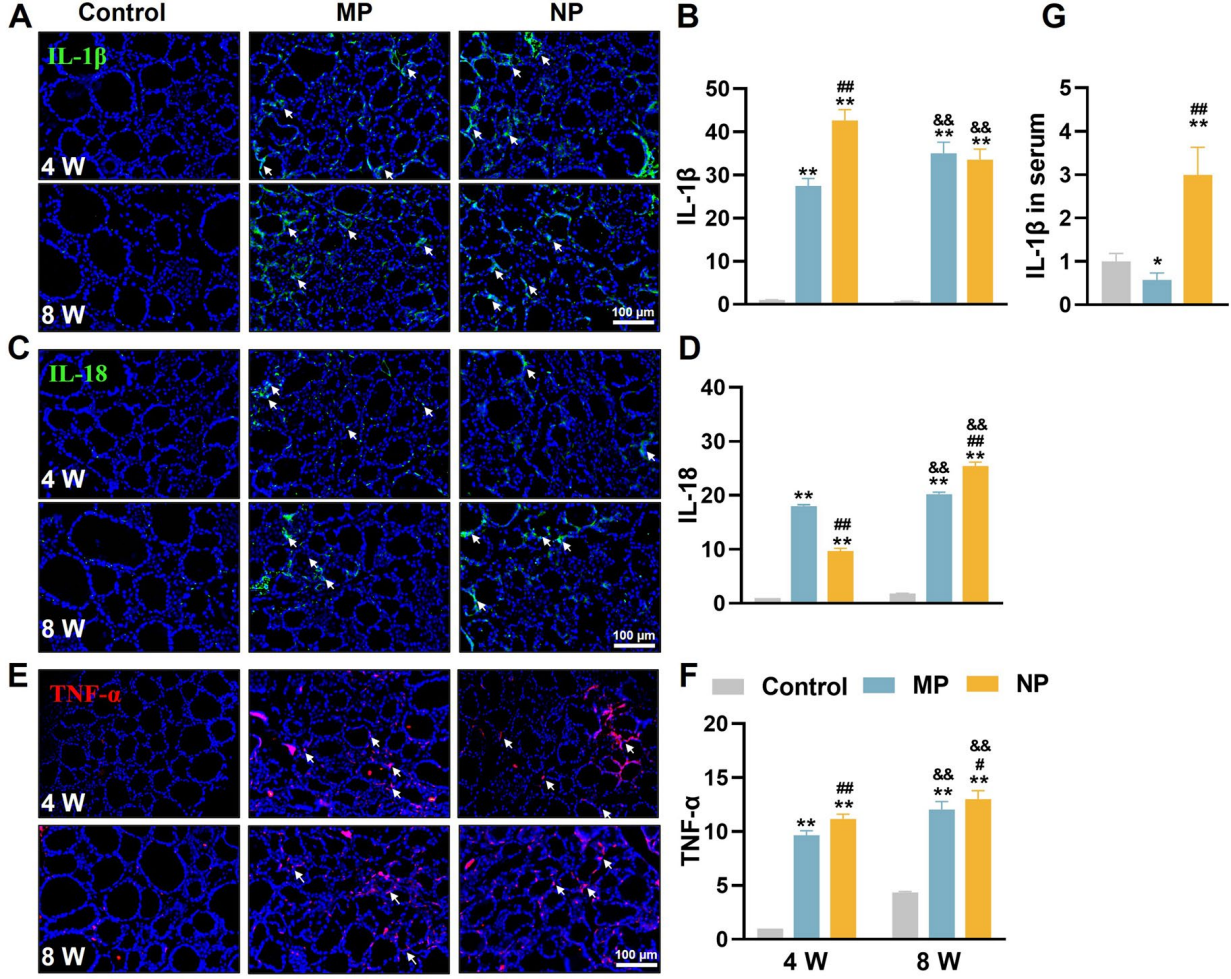
MNP exposure triggers inflammatory responses in thyroid tissue of mice. (A, C, E) Representative IF images showing the expression of IL-1β (green), IL-18 (green), and TNF-α (red) in thyroid at 4 and 8 weeks of MNP exposure. All sections were counterstained with DAPI (blue) to visualize cell nuclei. White arrows indicate positive expression of inflammatory cytokines in thyroid tissue. (B, D, F) Quantitative analysis of IL-1β (B), IL-18 (D), and TNF-α (F) expression levels in thyroid following 4 and 8 weeks of MNP exposure. The integrated optical density value in regions of interest were calculated using Image-Pro Plus software 6.0. (G) Serum IL-1β concentration determined by ELISA after 8 weeks of MNP exposure. Data are presented as mean ± SD (*n* = 6 per group). *p < 0.05, **p < 0.01 (versus control group at the same exposure time point); ^#^*p* < 0.05, ^##^*p* < 0.01 (versus MP group at the same exposure time point); ^&&^*p* < 0.01 (versus 4-week exposure group within the same treatment group).

To assess the inflammatory consequences of MNP exposure, we examined the expression of key pro-inflammatory cytokines (IL-1β, IL-18, and TNF-α) in thyroid follicular cells using immunofluorescence analysis using IF analysis. We found that MNP intervention significantly enhanced all three inflammatory cytokines in thyroid follicular cells, with distinct temporal patterns and particle size-dependent patterns (Figure 3A–F). IL-1β showed the most dramatic response, with 27.5-fold and 42.7-fold increases in MP and NP groups at 4 weeks, respectively. By 8 weeks, both treatments reached similar elevation levels (35.0–33.5 fold), suggesting convergent inflammatory responses over time (Figure 3B). IL-18 expression showed an inverse initial pattern, with MP inducing higher levels at 4 weeks (18.0-fold vs. 9.7-fold), but prolonged exposure reversed this relationship, with NP treatment resulting in significantly higher IL-18 levels at 8 weeks (25.4-fold vs. 20.2-fold for MP) (Figure 3D). TNF-α expression consistently favored NP-induced responses at both time points, with both treatments showing progressive increases from 4 weeks (9.7–11.2 fold) to 8 weeks (12.0–13.0 fold) (Figure 3E,F).

Interestingly, serum IL-1β analysis revealed differential effects of particle size on systemic inflammation. While NP exposure led to a 3.0-fold increase in circulating IL-1β compared to controls, MP treatment resulted in a reduction to 57.4% of control levels (Figure 3G), suggesting differential effects of particle size on systemic versus local inflammatory responses.

These findings demonstrate that MNP exposure triggers significant inflammatory responses in thyroid tissue, with NP generally eliciting stronger effects than MP. The time-dependent increases in inflammatory markers further indicate progressive inflammatory reactions during prolonged exposure to plastic particles, particularly NP.

### Exposure to MNP contributes to pyroptosis in thyroid cells

**Figure 4.**
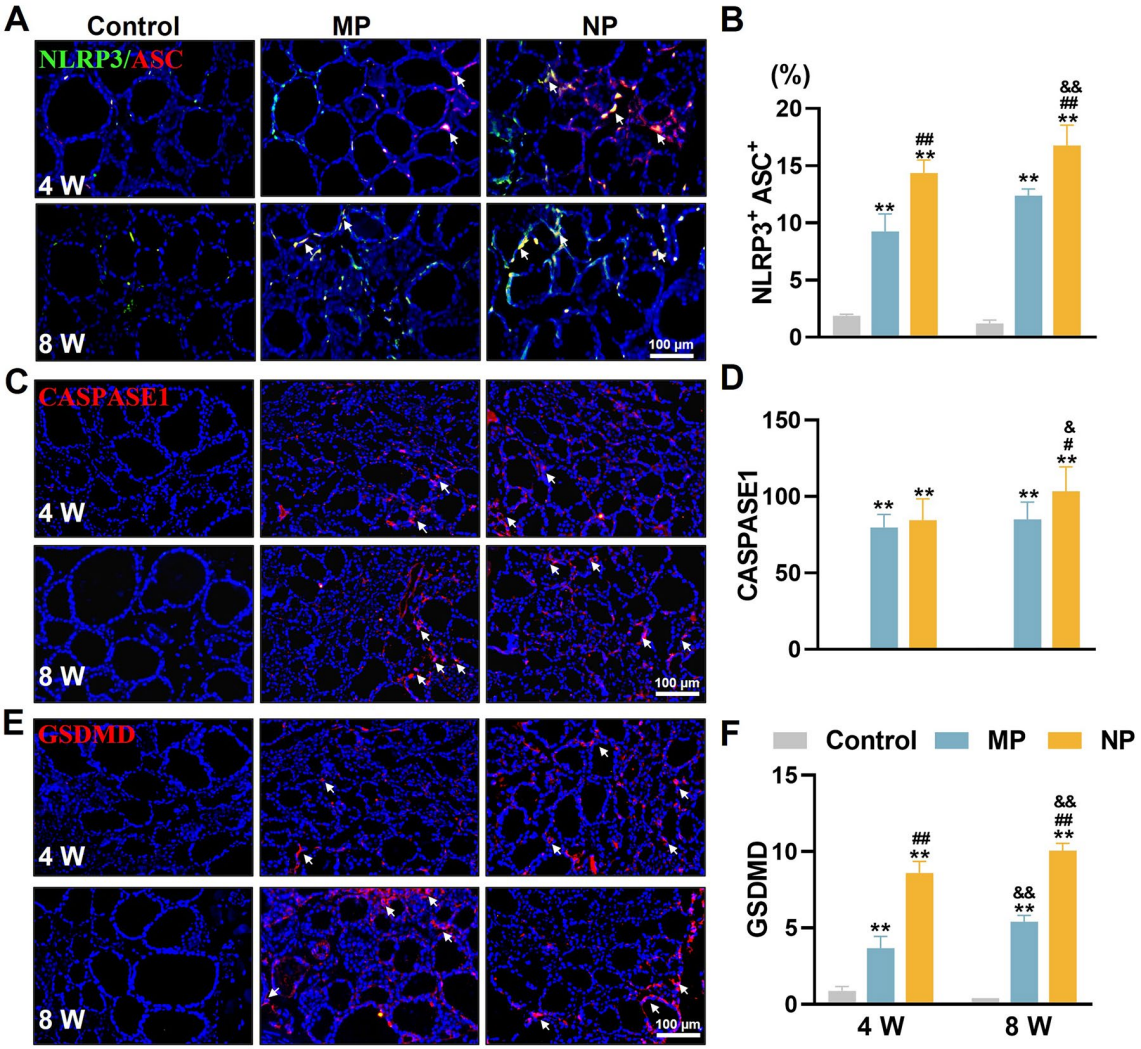
MNP exposure contributes to thyroid follicular cell pyroptosis in mice. (A) Representative IF images showing the colocalization of NLRP3 (green) and ASC (red) in thyroid at 4 and 8 weeks of MNP exposure. All sections were counterstained with DAPI (blue) to visualize cell nuclei. White arrows indicate both positive expression of NLRP3 and ASC in thyroid tissue. (B) Quantitative analysis of percentage of NLRP3^+^ASC^+^ cells in thyroid tissue following 4 and 8 weeks of MNP exposure. (C, E) Representative IF images showing the expression of CASPASE1 (red), and GSDMD (red) in thyroid at 4 and 8 weeks of MNP exposure. All sections were counterstained with DAPI (blue) to visualize cell nuclei. White arrows indicate positive expression of pyroptosis markers in thyroid tissue. (D, F) Quantitative analysis of CASPASE1, and GSDMD expression levels in thyroid tissue following 4 and 8 weeks of MNP exposure. The integrated optical density value in regions of interest were calculated using Image-Pro Plus software 6.0. Data are presented as mean ± SD (*n* = 6 per group). **p < 0.01 (versus control group at the same exposure time point); ^#^*p* < 0.05, ^##^*p* < 0.01 (versus MP group at the same exposure time point); ^&^*p* < 0.05, ^&&^*p* < 0.01 (versus 4-week exposure group within the same treatment group).

Given the substantial increases in IL-1β and IL-18 production in thyroid tissues following MNP exposure, we investigated whether MNP exposure activated pyroptotic cell death pathways by examining key pyroptosis mediators (NLRP3, CASPASE1, and GSDMD) in thyroid follicular cells using IF analysis. As shown in Figure 4A–F, both MP and NP exposure consistently markedly increased all three pyroptosis markers, with NP consistently inducing stronger responses. Importantly, the percentage of NLRP3^+^ASC^+^ cells, which represents the successful assembly of the NLRP3 inflammasome complex, showed sustained elevation in both groups, with MP inducing 5.0–10.2 fold increases at 4 and 8 weeks, respectively, while NP caused progressive increases from 7.7-fold to 13.8-fold (Figure 4B). CASPASE1, the central executioner of pyroptosis, demonstrated robust activation in both treatment groups. MP exposure resulted in stable 79.5–85.0-fold increases across time points, while NP treatment showed progressive elevation from 84.4-fold to 103.3-fold (Figure 4D). GSDMD, the terminal effector of pyroptotic cell death, exhibited time-dependent increases in both groups. MP treatment induced modest but significant increases (3.7-fold to 5.4-fold), while NP exposure resulted in more pronounced elevation (8.6-fold to 10.1-fold) (Figure 4F). These findings indicate that MNP exposure stimulates the excessive production of inflammatory cytokines primarily through the activation of NLRP3-mediated pyroptosis in thyroid follicular cells, with NP demonstrating stronger and more sustained pyroptotic responses than MP.

### MNP exposure activates NF-κB signaling pathway in thyroid follicular cells

**Figure 5.**
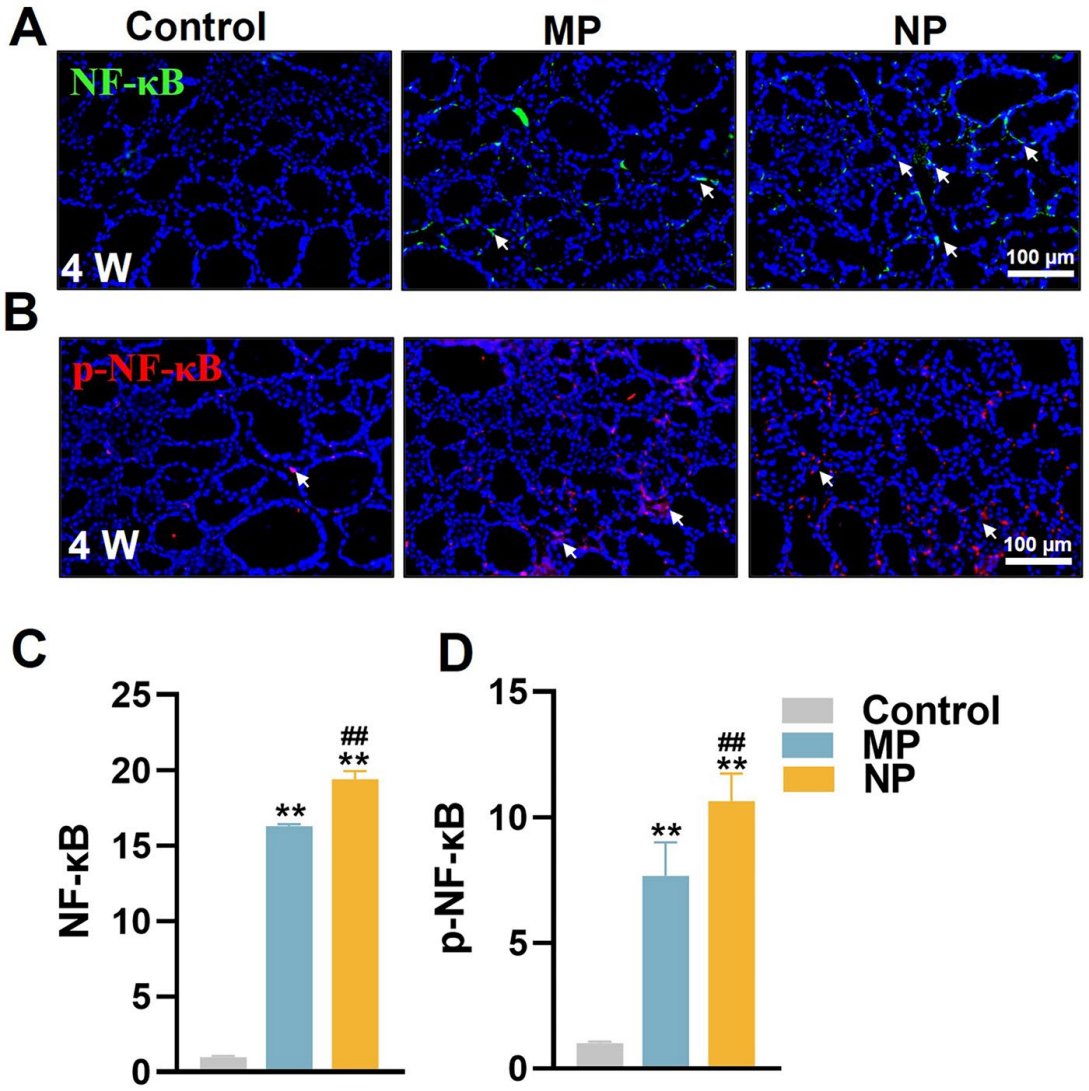
MNP exposure activates NF-κB signaling pathway in thyroid follicular cells of mice. (A, B) Representative IF images showing p-NF-κB (red) and NF-κB (green) expression in thyroid at 4 weeks of MNP exposure. All sections were counterstained with DAPI (blue) to visualize cell nuclei. White arrows indicate positive expression of NF-κB signaling markers in thyroid tissue. (C, D) Quantitative analysis of p-NF-κB and NF-κB expression levels in thyroid. The integrated optical density value in regions of interest were calculated using Image-Pro Plus software 6.0. Data are presented as mean ± SD (*n* = 6 per group). **p < 0.01 (versus control group at the same exposure time point); ^##^*p* < 0.01 (versus MP group at the same exposure time point).

To elucidate the potential mechanism underlying MP and NP-induced inflammatory and pyroptosis responses in thyroid follicular cells, we detected the expression and activation of NF-κB, a key transcription factor involved in inflammatory responses and pyroptosis regulation, using IF analysis. We found that both MP and NP exposure significantly increased total NF-κB (16.3-fold vs. 19.4-fold, respectively) and its phosphorylated form (p-NF-κB) (7.7-fold vs. 10.6-fold, respectively) after 4 weeks treatment, with consistently higher levels in the NP group compared to the MP group (Figure 5A–D), indicating more potent activation of the NF-κB signaling pathway by NP compared to MP. Taken together, these results suggest that MNP exposure activates the NF-κB signaling pathway in thyroid follicular cells in a particle size-dependent manner, providing a mechanistic link between MNP exposure and the subsequent inflammatory, pyroptotic, and apoptotic responses observed in thyroid tissue. The enhanced NF-κB activation, which considered to be canonical upstream signaling pathway to transcriptional enhance NLRP3, may induce pyroptosis initiation, ultimately contributing to thyroid dysfunction.

## Discussion

**Figure 6.**
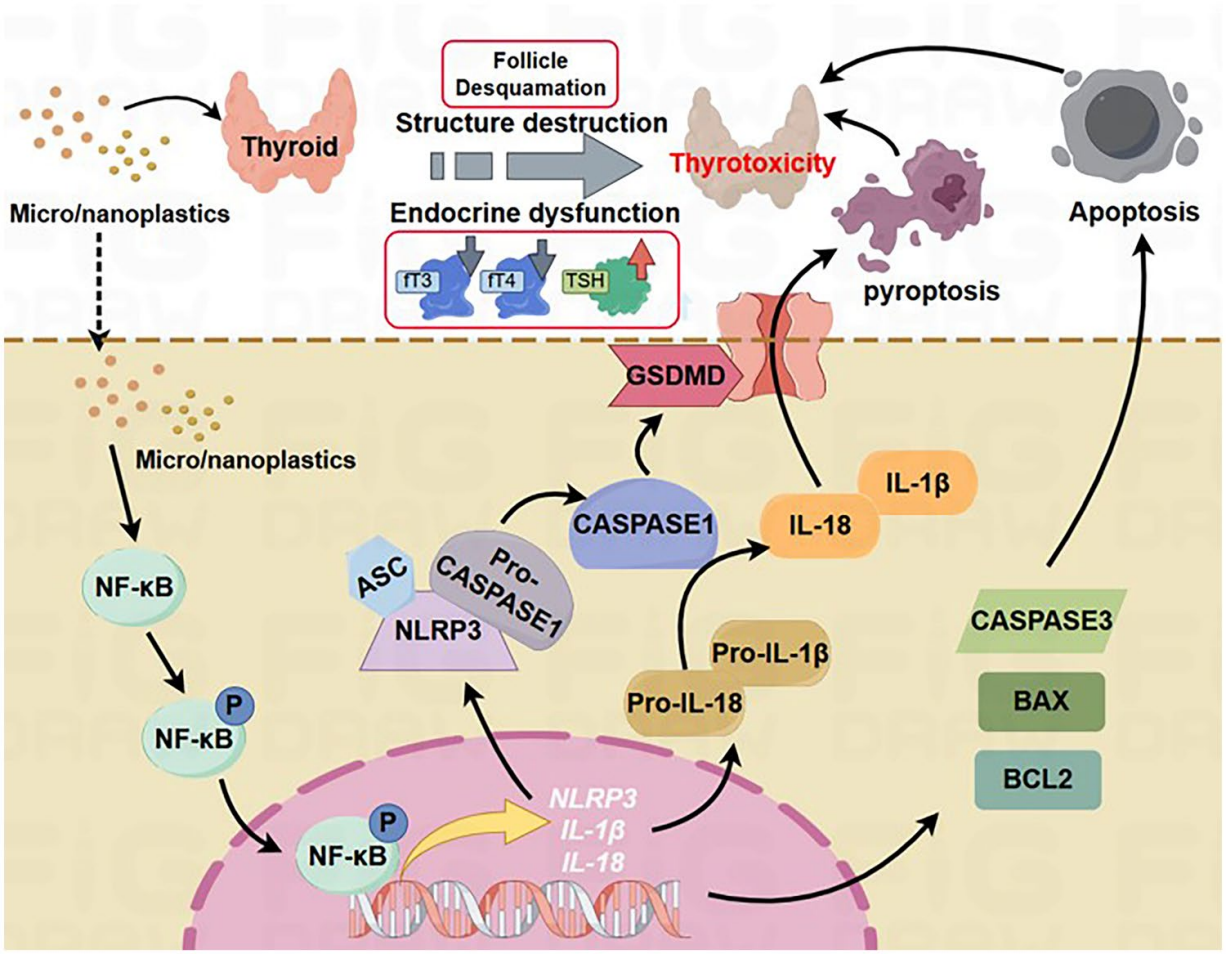
Schematic working model of MNP exposure-induced thyrotoxicity through NF-κB signaling-mediated thyroid follicular cell pyroptosis.

MNP contamination has emerged as a critical environmental health concern, with accumulating evidence documenting multi-organ toxicity across various biological systems [35–37]. While recent studies have identified associations between MNP exposure and thyroid dysfunction in aquatic species and rodent models [18,19,38,39], the precise molecular mechanisms underlying MNP-induced thyrotoxicity have remained enigmatic. In this study, to unravel the underlying mechanism of MNP exposure-induced thyrotoxicity, male mice were administered MP (5 μm) and NP (50 nm) at a dosage of 30 mg/kg for durations of 4 and 8 weeks, respectively. Our findings revealed that both MP and NP exposure induces severe thyroid architectural disruption, endocrine dysfunction, and inflammatory cytokine release, with NP exhibiting enhanced toxicity due to superior bioavailability and cellular penetration capacity. More importantly, MNP exposure triggers thyroid follicular cell pyroptosis through NF-κB-mediated NLRP3 inflammasome activation, fundamentally advancing our understanding of plastic particle toxicology (Figure 6). Our identification of the NF-κB/NLRP3/pyroptosis axis as the central mechanism underlying MNP-induced thyrotoxicity represents a paradigm shift with significant implications for environmental health policy and NP exposure risk assessment.

Human exposure to MNP has been quantified in recent studies, with adult weekly intake estimates ranging from 0.1 to 5 grams, corresponding to approximately 2–90 mg/kg daily when extrapolated to murine models [40]. The 30 mg/kg dosage employed in our study falls within this range and has been extensively validated in MNP toxicity research [16,32,33]. For instance, 35-day exposure to 30 mg/kg MP induced ovarian inflammation and compromised oocyte quality in mice [16]. Similarly, 28-day exposure to 30 mg/kg 4 μm MP disrupted the blood-testis barrier and impaired spermatogenesis [33], while 8-week exposure to 30 mg/kg 50 nm NP resulted in oxidative damage, insulin resistance, and pancreatic β-cell dysfunction [32]. These precedents support the environmental relevance and toxicological validity of our experimental design.

The relationship between particle size and biological toxicity represents a fundamental principle in nanotoxicology, with smaller particles generally exhibiting enhanced bioavailability and toxicological potency [37]. MNP uptake occurs primarily through gastrointestinal absorption, whose absorption efficiency is inversely correlated with particle diameter [41]. Jani and colleagues demonstrated that smaller MNP may exhibit higher absorption rates and more efficient cellular uptake compared to larger particles [42]. The size-dependent tissue distribution and cellular interaction of MNP influence their capacity to traverse biological membranes and accumulate in target organs [43].

Current evidence indicates that while MNP <150 µm can cross the gastrointestinal tract, particles <10 µm are capable of traversing biological barriers including the blood–brain barrier and even placenta, thereby increasing secondary tissue accumulation in organs such as the liver and brain [44–46]. For the reasons, multiple studies employ 5‑µm PS‑MPs to investigate liver, cardiotoxicity and multi‑organ toxicity [25,47,48]. Furthermore, MNP < 5 µm can be internalized by macrophages and subsequently transported through systemic circulation to the spleen and other organs [49]. A recent multiple species study used 50 nm PS‑NPs at mouse doses of 5–30 mg/kg to track distribution and oxidative stress [50]. Nanoparticles of this size can readily penetrate cell membranes, enter the circulation, and reach distant organs, thereby eliciting more serious cellular‑level toxicity such as oxidative stress and apoptosis. The enhanced toxicity of smaller particles is attributed to their greater surface area-to-volume ratio, which facilitates the increased release of endocrine-disrupting chemicals and other harmful compounds [43]. Our findings align with these principles, demonstrating that NP exposure resulted in more severe thyroid architectural damage and endocrine dysfunction compared to MP treatment. This size-dependent toxicity likely reflects enhanced absorption, superior transmembrane penetration capacity, and preferential accumulation of NP in thyroid follicular cells, leading to amplified thyrotoxic effects.

The thyroid gland plays a central role in growth, development, and metabolism through the regulation of thyroid hormones, such as TSH, T_3_ and T_4_ [51]. These hormonal biomarkers serve as reliable markers of thyroid endocrine function in both clinical and experimental settings [52]. Chronic exposure to environmental contaminants, including plastic particles, has been consistently associated with thyroid endocrine disruption, manifested as imbalances in serum T_4_, T_3_, and/or TSH levels [53]. For instance, 5-week exposure to 10 mg/kg 50 nm NP in adult male Wistar rats significantly suppressed serum fT_3_ and fT_4_ levels while elevating TSH concentrations [19]. Furthermore, exposure to 20 and 200 μg/L 10 μm MP for 21 days in juvenile Japanese flounder exacerbated polychlorinated biphenyls-induced thyroid disruption, evidenced by decreased T_3_ and T_4_ levels and altered HPT axis gene expression [39]. Our results corroborate these findings, demonstrating that both MP (5 μm) and NP (50 nm) exposure significantly reduced serum fT_4_ and fT_3_ levels while elevating TSH concentrations. The more pronounced hormonal disruption observed with NP exposure likely reflects the enhanced bioavailability and tissue penetration of smaller particles. The compensatory TSH elevation represents a classical negative feedback response to diminished thyroid hormone production, though the differential TSH response between MP and NP groups suggests additional complexity in HPT axis regulation [54]. The ability of NP to penetrate the blood-brain barrier may contribute to hypothalamic-pituitary dysfunction, further compromising the negative feedback mechanisms governing thyroid hormone homeostasis [43].

Thyroid hormone synthesis occurs within follicular epithelial cells, with hormone storage in colloid-filled follicles; therefore, thyroid tissue architecture directly influences hormonal production capacity [55]. Our histopathological analysis revealed significant reductions in follicle diameter and epithelial cell thickness following MNP exposure, accompanied by increased follicle density indicative of compensatory hyperplasia. These structural alterations provide a mechanistic basis for the observed endocrine dysfunction, with NP exposure causing more severe architectural disruption than MP treatment. Based on the widespread use of plastics and the lack of thorough degradation technology, the accumulation of MNP and the degradation to smaller particles will raise the risk of hypothyroidism.

Mounting evidence demonstrates that MNP exposure triggers robust inflammatory responses characterized by elevated inflammatory cytokine levels in both systemic circulation and target organs. Previous studies have shown that MP exposure in C57BL/6J mice resulted in hepatic inflammation with increased expression of IFN-γ, TNF-α, IL-1β, IL-6, and IL-33 [56]. Likewise, 28-day MP exposure significantly elevated serum inflammatory cytokines and renal gene expression in female SD rats [46]. In addition, MPs trigger macrophage extracellular trap formation *via* ROS‑driven mitochondrial and lysosomal damage to induce the liver inflammation [47]. Inflammatory cytokines, particularly IL-1β and TNF-α, have been implicated in thyroid epithelial damage in autoimmune thyroid diseases, while elevated IL-1β and IL-18 levels contribute to thyrocyte destruction in Hashimoto’s thyroiditis [57]. Our findings demonstrate pronounced infiltration of IL-1β, IL-18, and TNF-α in thyroid tissue following MNP exposure, concurrent with follicular structural destruction. A more severe inflammatory response observed with NP exposure aligns with previous reports showing size-dependent inflammatory effects, with smaller particles inducing more pronounced tissue damage [58].

Our study provides novel evidence that pyroptosis represents a critical potential mechanism underlying MNP-induced thyrotoxicity. While apoptotic cell death was observed, the magnitude of pyroptotic protein upregulation (hundreds-fold increases) far exceeded that of apoptotic markers (20-fold increases), suggesting pyroptosis as the predominant cell death pathway. This distinction is mechanistically important, as pyroptosis, unlike apoptosis, is inherently inflammatory and promotes cytokine release. The robust activation of NLRP3, CASPASE1, and GSDMD in thyroid tissue following MNP exposure, coupled with the dramatic elevation of IL-1β and IL-18, confirms pyroptotic pathway engagement. Previous studies have documented MNP-induced pyroptosis in multiple organs, including lung, heart, ovary, and liver, resulting in inflammatory factor release and tissue damage [22,23,25,59]. Our findings extend these observations to thyroid tissue and demonstrate the size-dependent nature of pyroptotic responses, with NP inducing stronger and more sustained effects than MP. Moreover, pyroptosis-related-proteins especially CASPASE1 were greatly elevated in the early stage of microplastics exposure, suggesting that these proteins have the potential to become biomarkers for clinical diagnosis of thyroid injury caused by MNP.

The NF-κB signaling pathway serves as a master regulator of inflammatory responses, apoptosis, and pyroptosis in thyroid cells, with aberrant activation contributing to hypothyroidism and various thyroid pathologies [60,61]. Recent evidences indicates that NF-κB activation triggers thyroid cell pyroptosis and promotes inflammatory cytokine expression, while NF-κB inhibition prevents pyroptotic cell death [27,60]. Our demonstration of significant NF-κB and p-NF-κB upregulation following MNP exposure, with NP inducing greater activation than MP, provides a potential mechanistic insight into the upstream regulation of pyroptotic responses. This size-dependent NF-κB activation aligns with previous reports of MNP-induced NF-κB signaling in testicular, renal, and intestinal tissues [30,31,62,63], supporting a conserved mechanism of plastic particle toxicity across organ systems.

Several limitations warrant acknowledgment in this study. Firstly, this research was carried out exclusively on male mice to ensure hormonal consistency, minimize variability, and follow historical precedent focused on initial mechanistic discovery. But thyroid disorders particularly autoimmune conditions like Hashimoto’s thyroiditis linked to inflammation and pyroptosis, exhibit a strong female predominance in humans. It severely constrains the study’s translational impact and produces findings relevant only to half the population, potentially overlooking heightened female susceptibility. Our future studies will include both sexes to fully understand the toxicological profile of MNP on the thyroid. Second, we only used a single exposure level of MNP and did not quantify MNP concentrations in serum or thyroid tissue, which limits our ability to establish dose-response relationships and correlate tissue burden with toxicological outcomes. In the future, it is necessary to correlate the exposure level of MNP with thyroid function tests and MNP concentrations in serum or thyroid tissue in a clinical cohort. Besides, the evidence of NF-κB activation, pyroptosis triggering and thyroid destruction were correlative. The causality remains to be confirmed through future functional inhibition experiments. Moreover, to enhance the robustness of our findings, incorporating additional quantitative approaches is important. But complete separation of thyroid gland from adjacent structures is too technically difficult to achieve without contamination. To justify the mechanisms involved, we considered to incorporating laser capture microdissection coupled with the targeted proteomics or single-cell RNA sequencing in future investigations to provide more comprehensive molecular profiling while circumventing tissue purity limitations. Third, our investigation focused exclusively on thyroid tissue without a comprehensive assessment of HPT axis function, which would provide a more complete understanding of MNP effects on thyroid hormone homeostasis. Fourth, the reversibility of MNP-induced thyrotoxicity following exposure cessation remains unexplored, despite evidence suggesting recovery potential in other organ systems [58]. Future investigations should address these limitations through both sexes’ studies, comprehensive exposure assessment, HPT axis evaluation, and recovery studies to provide a complete and clinically relevant understanding of MNP-induced thyrotoxicity and fulfill the goals of environmental health protection. Additionally, mechanistic studies examining the role of oxidative stress, autophagy, and other cellular pathways in MNP-induced thyrotoxicity would enhance our understanding of plastic particle toxicology. Moreover, while no overt signs of gavage-related distress or significant weight loss were observed, we acknowledge that daily gavage could potentially influence short-term food and water intake. Future studies incorporating detailed metabolic cage monitoring would be valuable to fully dissect any confounding effects of the administration procedure from the direct toxicity of the particles.

## Conclusions

In summary, our findings illustrate that MNP exposure exerts thyrotoxicity by disrupting thyroid structure and endocrine function. Specifically, MP and NP facilitate NLRP3-mediated follicular cell pyroptosis accompanied by activating NF-κB signaling, with NP demonstrating superior toxicological potency compared to MP. Our findings establish pyroptosis as an important potential mechanism underlying plastic particle-induced endocrine disruption, offering novel insights into environmental plastic toxicology and highlighting the urgent need for comprehensive exposure risk assessment, particularly for nanoscale plastic particles that exhibit enhanced bioavailability and tissue penetration capacity. Future research should examine pyroptosis mechanisms under varying concentrations, extended exposure and both sexes to further assess MNPs’ ecological and health risks of thyroid.

## Supplementary Material

Supplemental Material

Supplemental Material

Supplemental Material

## Data Availability

All the data are available within this manuscript or from the corresponding author upon reasonable request.

## Abbreviations

MNP: Micro/nanoplastics
MP: Microplastics
NP: Nanoplastics
T_4_: Thyroxine
T_3_: Triiodothyronine
TSH: Thyroidstimulated hormone
fT_4_: Free Thyroxine
fT_3_: Free Triiodothyronine
NLRP3: NOD-like receptor protein 3
IL-1β: Interleukin 1β
IL-18: Interleukin 18
GSDMD: Gasdermin D
CASPASE1: Cysteinyl aspartate specific proteinase 1
BCL2: B-cell lymphoma-2
BAX: BCL2-associated X protein
CASPASE3: Cysteinyl aspartate specific proteinase 3
TNF-α: Tumor necrosis factor-alpha
NF-κB: Nuclear factor kappa-B
ELISA: Immunosorbent assay
IF: Immunofluorescence
DF: Diameter of the follicle
HE: Hematoxylin–Eosin
PAS: Periodic Acid Schiff
