# Supplementary material for: Micro/nanoplastics induce thyroid follicular cell pyroptosis to trigger thyrotoxicity by activating NF-κB signaling
Source: Ann Med. 2026 Feb 17;58(1):2624175. doi: 10.1080/07853890.2026.2624175 (PMC12915396; doi:10.1080/07853890.2026.2624175)
Supplement: Supplemental Material [file IANN_A_2624175_SM2410.docx]

**Supplementary Figure**

Figure S1. (A) The SEM image, (B) Polydispersity Index (Pi) and Zeta potential, and (C) particle size distribution of the MNP.
